# Perchlorato[*N*,*N*,*N*′,*N*′-tetra­kis(2-pyridyl­meth­yl)cyclo­hexane-1,2-diamine]manganese(II) perchlorate

**DOI:** 10.1107/S1600536808025804

**Published:** 2008-08-16

**Authors:** In-Chul Hwang, Kwang Ha

**Affiliations:** a Department of Chemistry, Pohang University of Science and Technology, Pohang 790-784, Republic of Korea; b School of Applied Chemical Engineering, Research Institute of Catalysis, Chonnam National University, Gwangju 500-757, Republic of Korea

## Abstract

The asymmetric unit of the title compound, [Mn(ClO_4_)(C_30_H_34_N_6_)]ClO_4_, consists of a cationic [Mn(ClO_4_)(C_30_H_34_N_6_)]^+^ complex and a perchlorate anion. In the complex, the Mn^2+^ ion is seven-coordinated in an approximately penta­gonal–bipyramidal environment by six N atoms from the hexa­dentate *N*,*N*,*N*′,*N*′-tetra­kis(2-pyridylmeth­yl)­cyclo­hexane-1,2-diamine (tpdach) ligand and one O atom from a perchlorate anion. The complex displays inter­molecular π–π inter­actions between adjacent pyridine rings (centroid-to-centroid distance 4.133 Å). Moreover, there are weak intra- and inter­molecular C—H⋯O hydrogen bonds. The Cl atom and two O atoms of the perchlorate are disordered, with a site-occupancy factor of 0.59 (2) for the major component.

## Related literature

For details of some other tpdach complexes, see: Hwang & Ha (2006 ▶); McCusker *et al.* (1993 ▶). For the synthesis of the ligand, see: Toftlund & Yde-Anderson (1981 ▶).
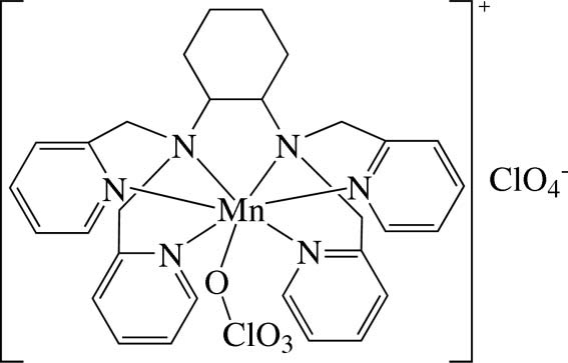


## Experimental

### 

#### Crystal data

[Mn(ClO_4_)(C_30_H_34_N_6_)]ClO_4_
*M* *_r_* = 732.47
Monoclinic, 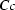

*a* = 14.223 (5) Å
*b* = 14.121 (5) Å
*c* = 16.504 (6) Å
β = 105.987 (6)°
*V* = 3186.6 (19) Å^3^
*Z* = 4
Mo *K*α radiation
μ = 0.64 mm^−1^
*T* = 293 (2) K
0.25 × 0.25 × 0.20 mm

#### Data collection

Bruker SMART 1000 CCD diffractometer
Absorption correction: multi-scan (*SADABS*; Bruker, 2000 ▶) *T*_min_ = 0.690, *T*_max_ = 0.879
9661 measured reflections
5412 independent reflections
4446 reflections with *I* > 2σ(*I*)
*R*_int_ = 0.020

#### Refinement

*R*[*F* ^2^ > 2σ(*F* ^2^)] = 0.039
*wR*(*F* ^2^) = 0.093
*S* = 0.96
5412 reflections
452 parameters
2 restraints
H-atom parameters constrained
Δρ_max_ = 0.39 e Å^−3^
Δρ_min_ = −0.29 e Å^−3^
Absolute structure: Flack (1983 ▶), with 1804 Friedel pairs
Flack parameter: 0.007 (16)

### 

Data collection: *SMART* (Bruker, 2000 ▶); cell refinement: *SAINT* (Bruker, 2000 ▶); data reduction: *SAINT*; program(s) used to solve structure: *SHELXS97* (Sheldrick, 2008 ▶); program(s) used to refine structure: *SHELXL97* (Sheldrick, 2008 ▶); molecular graphics: *ORTEP-3* (Farrugia, 1997 ▶) and *PLATON* (Spek, 2003 ▶); software used to prepare material for publication: *SHELXL97*.

## Supplementary Material

Crystal structure: contains datablocks global, I. DOI: 10.1107/S1600536808025804/zl2134sup1.cif

Structure factors: contains datablocks I. DOI: 10.1107/S1600536808025804/zl2134Isup2.hkl

Additional supplementary materials:  crystallographic information; 3D view; checkCIF report

## Figures and Tables

**Table 1:** Hydrogen-bond geometry (Å, °)

*D*—H⋯*A*	*D*—H	H⋯*A*	*D*⋯*A*	*D*—H⋯*A*
C7—H7⋯O1	0.93	2.41	3.006 (5)	122
C17—H17*A*⋯O2^i^	0.97	2.48	3.413 (5)	161
C18—H18⋯O8^ii^	0.98	2.47	3.436 (6)	170
C20—H20⋯O6	0.93	2.46	3.347 (6)	159
C25—H25⋯O1	0.93	2.46	3.038 (5)	121
C25—H25⋯O2	0.93	2.51	3.199 (5)	131
C28—H28⋯O8^iii^	0.93	2.48	3.243 (6)	139
C30—H30*A*⋯O3*A*^i^	0.97	2.55	3.434 (11)	152

Symmetry codes: (i) 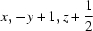; (ii) 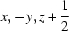; (iii) 
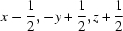
.

**Fig. 1.**
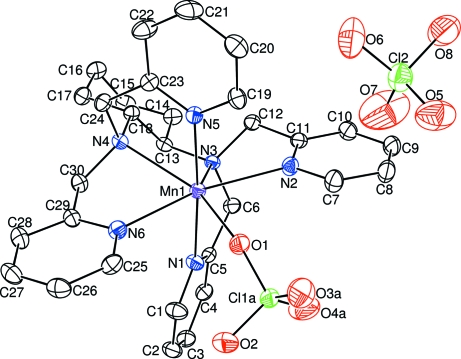
The structure of the title compound with the numbering scheme. Displacement ellipsoids are drawn at the 20% probability level. H atoms have been omitted for clarity, and only the major component of the disordered atoms Cl, O3 and O4 is shown.

**Fig. 2.**
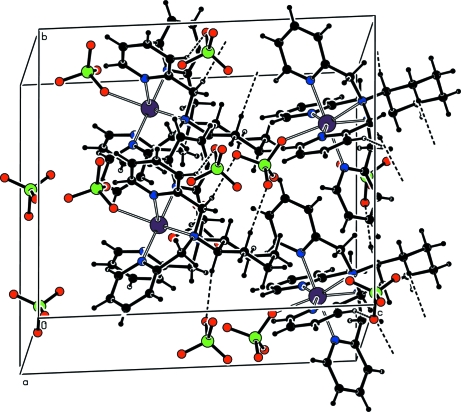
View of the unit-cell contents of the title compound. Hydrogen-bond interactions are drawn as dashed lines.

## supplementary crystallographic information

### Comment

The crystal structure of the title compound consists of a cationic complex, [Mn(ClO_4_)(C_30_H_34_N_6_)]^+^, and a ClO_4_^-^ anion (Fig. 1). In the cation, the Mn^2+^ ion is seven-coordinated by six N atoms from tpdach and one O atom from a ClO_4_^-^ ligand in an approximately pentagonal bipyramidal structure, in which the N2, N3, N4, N6 and O1 atoms form the pentagonal plane with the N1 and N5 atoms at the apices. In the analogous Fe^2+^ compound [Fe(C_30_H_34_N_6_)](ClO_4_)_2_ the Fe ion is six-coordinated in a distorted octahedral environment (McCusker *et al.* 1993). The apical N1—Mn1—N5 bond angle is 176.0 (1)°, and the Mn1—N1 and Mn1—N5 bond lengths are nearly equivalent (2.294 (3) Å and 2.273 (3) Å, respectively) and shorter than the Mn1—N bonds for N2, N3, N4 and N6 in the equatorial plane (2.388 (3) Å to 2.422 (3) Å). Within the plane, the chelating angles lie in the range of 70.76 (9) to 74.13 (9)° and the O1—Mn1—N bond angles for N2 and N6 are 78.9 (1)° and 78.1 (1), respectively. The complex displays intermolecular π–π interactions between adjacent pyridine rings. The shortest distance between *Cg*1 (the centroid of six-membered ring N2–C11) and *Cg*2*^a^* (ring N6–C29, symmetry code a: 1/2 + *x*,-1/2 + *y*,*z*) is 4.133 Å, and the dihedral angle between the ring planes is 6.59°. The C10···C27*^a^* and C11···C27*^a^* distances are 3.469 Å and 3.491 Å, respectively. Moreover, there are intra- and intermolecular hydrogen bonds between the C and O atoms with d(C···O) = 3.006 Å to 3.436 Å (Fig. 2, Table 1).

### Experimental

*N*,*N*,*N*',*N*'-Tetrakis(2-pyridylmethyl)cyclohexane-1,2-diamine (tpdach), C_30_H_34_N_6_, was synthesized according to a literature procedure (Toftlund & Yde-Anderson, 1981). Mn(ClO_4_)_2_.6H_2_O (0.61 g, 1.69 mmol) and tpdach (0.40 g, 0.84 mmol) in EtOH (10 ml) were stirred for 2 h at room temperature. The solvent was removed *in vacuo*, the residue recrystallized from acetone–ether and filtered, to give a dark yellow powder (0.44 g). Crystals suitable for X-ray analysis were obtained by slow evaporation from an acetone–ethyl acetate solution. MS (FAB): m/*z* 632, 634 (Mn(tpdach)(ClO_4_)^+^).

### Refinement

H atoms were positioned geometrically and allowed to ride on their respective carrier atoms [C—H = 0.93 (aromatic CH), 0.97 (CH_2_) or 0.98 Å (CH) and *U*_iso_(H) = 1.2*U*_eq_(C)]. The ClO_4_^-^ anions (particularly Cl1, O3 and O4) displayed relatively large displacement factors so that the anions appeared to be partially disordered. Atoms Cl1, O3 and O4 were thus modelled anisotropically as disordered over two sites, with a site occupancy factor of 0.59 (2) for the major component. Floating origin restraints were generated automatically by the program SHELXL97.

### Crystal data

**Table:**

[Mn(ClO_4_)(C_30_H_34_N_6_)]ClO_4_	*F*_000_ = 1516
*M**_r_* = 732.47	*D*_x_ = 1.527 Mg m^−^^3^
Monoclinic, *C**c*	Mo *K*α radiation λ = 0.71073 Å
Hall symbol: C -2yc	Cell parameters from 3537 reflections
*a* = 14.223 (5) Å	θ = 2.6–22.9º
*b* = 14.121 (5) Å	µ = 0.64 mm^−^^1^
*c* = 16.504 (6) Å	*T* = 293 (2) K
β = 105.987 (6)º	Stick, yellow
*V* = 3186.6 (19) Å^3^	0.25 × 0.25 × 0.20 mm
*Z* = 4	

### Data collection

**Table:**

Bruker SMART 1000 CCD diffractometer	5412 independent reflections
Radiation source: fine-focus sealed tube	4446 reflections with *I* > 2σ(*I*)
Monochromator: graphite	*R*_int_ = 0.020
*T* = 293(2) K	θ_max_ = 27.6º
φ and ω scans	θ_min_ = 2.1º
Absorption correction: multi-scan(SADABS; Bruker, 2000)	*h* = −11→18
*T*_min_ = 0.690, *T*_max_ = 0.879	*k* = −18→18
9661 measured reflections	*l* = −21→21

### Refinement

**Table:**

Refinement on *F*^2^	Hydrogen site location: inferred from neighbouring sites
Least-squares matrix: full	H-atom parameters constrained
*R*[*F*^2^ > 2σ(*F*^2^)] = 0.039	*w* = 1/[σ^2^(*F*_o_^2^) + (0.103*P*)^2^] where *P* = (*F*_o_^2^ + 2*F*_c_^2^)/3
*wR*(*F*^2^) = 0.093	(Δ/σ)_max_ = 0.001
*S* = 0.96	Δρ_max_ = 0.39 e Å^−^^3^
5412 reflections	Δρ_min_ = −0.29 e Å^−^^3^
452 parameters	Extinction correction: none
2 restraints	Absolute structure: Flack (1983), with 1804 Friedel pairs
Primary atom site location: structure-invariant direct methods	Flack parameter: 0.007 (16)
Secondary atom site location: difference Fourier map	

### Special details

**Table:**

Geometry. All e.s.d.'s (except the e.s.d. in the dihedral angle between two l.s. planes) are estimated using the full covariance matrix. The cell e.s.d.'s are taken into account individually in the estimation of e.s.d.'s in distances, angles and torsion angles; correlations between e.s.d.'s in cell parameters are only used when they are defined by crystal symmetry. An approximate (isotropic) treatment of cell e.s.d.'s is used for estimating e.s.d.'s involving l.s. planes.
Refinement. Refinement of *F*^2^ against ALL reflections. The weighted *R*-factor *wR* and goodness of fit *S* are based on *F*^2^, conventional *R*-factors *R* are based on *F*, with *F* set to zero for negative *F*^2^. The threshold expression of *F*^2^ > σ(*F*^2^) is used only for calculating *R*-factors(gt) *etc*. and is not relevant to the choice of reflections for refinement. *R*-factors based on *F*^2^ are statistically about twice as large as those based on *F*, and *R*- factors based on ALL data will be even larger.

### Fractional atomic coordinates and isotropic or equivalent isotropic displacement parameters (Å^2^)

**Table:**

	*x*	*y*	*z*	*U*_iso_*/*U*_eq_	Occ. (<1)
Mn1	0.15888 (5)	0.33639 (3)	0.36637 (4)	0.03769 (12)	
N1	0.2229 (2)	0.47899 (19)	0.42192 (18)	0.0450 (7)	
N2	0.2968 (2)	0.2727 (2)	0.32882 (19)	0.0494 (7)	
N3	0.2886 (2)	0.30494 (17)	0.49337 (17)	0.0385 (6)	
N4	0.0803 (2)	0.27813 (17)	0.46689 (15)	0.0362 (6)	
N5	0.0853 (2)	0.19852 (19)	0.31237 (16)	0.0399 (6)	
N6	0.0022 (2)	0.4106 (2)	0.34287 (18)	0.0465 (7)	
C1	0.1793 (3)	0.5634 (2)	0.3961 (3)	0.0597 (10)	
H1	0.1232	0.5641	0.3510	0.072*	
C2	0.2147 (3)	0.6482 (2)	0.4339 (3)	0.0583 (10)	
H2	0.1831	0.7048	0.4144	0.070*	
C3	0.2968 (3)	0.6477 (2)	0.5004 (2)	0.0549 (9)	
H3	0.3217	0.7037	0.5276	0.066*	
C4	0.3421 (3)	0.5623 (2)	0.5264 (2)	0.0483 (8)	
H4	0.3984	0.5606	0.5713	0.058*	
C5	0.3045 (2)	0.4795 (2)	0.4861 (2)	0.0393 (7)	
C6	0.3564 (3)	0.3869 (2)	0.5087 (2)	0.0469 (8)	
H6A	0.4029	0.3790	0.4758	0.056*	
H6B	0.3928	0.3881	0.5677	0.056*	
C7	0.3158 (3)	0.2794 (3)	0.2543 (2)	0.0605 (11)	
H7	0.2675	0.3039	0.2091	0.073*	
C8	0.4026 (3)	0.2522 (3)	0.2405 (3)	0.0668 (11)	
H8	0.4129	0.2583	0.1874	0.080*	
C9	0.4742 (3)	0.2156 (3)	0.3069 (3)	0.0619 (10)	
H9	0.5345	0.1978	0.3000	0.074*	
C10	0.4550 (3)	0.2060 (3)	0.3832 (3)	0.0567 (9)	
H10	0.5020	0.1806	0.4288	0.068*	
C11	0.3656 (3)	0.2342 (2)	0.3920 (2)	0.0446 (8)	
C12	0.3408 (3)	0.2206 (2)	0.4749 (2)	0.0472 (8)	
H12A	0.3001	0.1650	0.4717	0.057*	
H12B	0.4004	0.2111	0.5199	0.057*	
C13	0.2477 (2)	0.2910 (2)	0.5668 (2)	0.0401 (7)	
H13	0.2314	0.3540	0.5838	0.048*	
C14	0.3198 (3)	0.2466 (3)	0.6443 (2)	0.0504 (9)	
H14A	0.3382	0.1840	0.6299	0.060*	
H14B	0.3785	0.2850	0.6608	0.060*	
C15	0.2752 (3)	0.2392 (3)	0.7173 (2)	0.0556 (10)	
H15A	0.3224	0.2120	0.7658	0.067*	
H15B	0.2580	0.3018	0.7327	0.067*	
C16	0.1864 (3)	0.1786 (3)	0.6929 (2)	0.0541 (10)	
H16A	0.1589	0.1722	0.7403	0.065*	
H16B	0.2039	0.1159	0.6779	0.065*	
C17	0.1106 (3)	0.2223 (3)	0.6182 (2)	0.0491 (9)	
H17A	0.0904	0.2833	0.6344	0.059*	
H17B	0.0534	0.1817	0.6023	0.059*	
C18	0.1528 (2)	0.2349 (2)	0.54187 (18)	0.0381 (7)	
H18	0.1686	0.1717	0.5251	0.046*	
C19	0.0988 (3)	0.1550 (3)	0.2434 (2)	0.0496 (8)	
H19	0.1363	0.1852	0.2129	0.060*	
C20	0.0597 (3)	0.0692 (3)	0.2171 (2)	0.0612 (11)	
H20	0.0707	0.0412	0.1694	0.073*	
C21	0.0044 (4)	0.0238 (3)	0.2606 (3)	0.0734 (14)	
H21	−0.0217	−0.0358	0.2440	0.088*	
C22	−0.0122 (4)	0.0680 (3)	0.3298 (2)	0.0645 (11)	
H22	−0.0505	0.0391	0.3601	0.077*	
C23	0.0290 (3)	0.1558 (2)	0.35356 (19)	0.0402 (7)	
C24	0.0067 (3)	0.2074 (2)	0.4259 (2)	0.0432 (8)	
H24A	−0.0560	0.2388	0.4054	0.052*	
H24B	0.0006	0.1613	0.4677	0.052*	
C25	−0.0532 (3)	0.4527 (3)	0.2737 (2)	0.0533 (9)	
H25	−0.0313	0.4530	0.2255	0.064*	
C26	−0.1411 (3)	0.4959 (3)	0.2703 (3)	0.0592 (10)	
H26	−0.1773	0.5251	0.2211	0.071*	
C27	−0.1740 (3)	0.4950 (3)	0.3403 (3)	0.0617 (11)	
H27	−0.2332	0.5234	0.3397	0.074*	
C28	−0.1184 (3)	0.4515 (3)	0.4115 (3)	0.0555 (9)	
H28	−0.1400	0.4495	0.4598	0.067*	
C29	−0.0304 (3)	0.4106 (2)	0.4119 (2)	0.0432 (8)	
C30	0.0319 (3)	0.3630 (2)	0.4887 (2)	0.0441 (8)	
H30A	0.0812	0.4071	0.5195	0.053*	
H30B	−0.0083	0.3450	0.5250	0.053*	
O1	0.1329 (2)	0.3954 (2)	0.23005 (16)	0.0643 (8)	
O2	0.0969 (4)	0.5516 (2)	0.1871 (3)	0.1098 (14)	
Cl1A	0.1592 (6)	0.4718 (3)	0.1811 (5)	0.0695 (14)	0.589 (18)
O3A	0.1337 (14)	0.4370 (8)	0.0960 (6)	0.122 (5)	0.589 (18)
O4A	0.2592 (6)	0.4953 (6)	0.2141 (11)	0.134 (5)	0.589 (18)
Cl1B	0.1171 (11)	0.4580 (8)	0.1590 (6)	0.091 (3)	0.411 (18)
O3B	0.042 (2)	0.4334 (16)	0.0973 (10)	0.214 (13)	0.411 (18)
O4B	0.2022 (18)	0.4735 (14)	0.1375 (18)	0.147 (12)	0.411 (18)
Cl2	0.16080 (10)	0.07166 (10)	0.01224 (8)	0.0782 (3)	
O5	0.1508 (5)	0.1150 (3)	−0.0657 (3)	0.1315 (16)	
O6	0.0759 (4)	0.0230 (4)	0.0224 (3)	0.1293 (16)	
O7	0.2066 (7)	0.1294 (5)	0.0740 (4)	0.221 (4)	
O8	0.2213 (5)	−0.0077 (4)	0.0100 (5)	0.198 (3)	

### Atomic displacement parameters (Å^2^)

**Table:**

	*U*^11^	*U*^22^	*U*^33^	*U*^12^	*U*^13^	*U*^23^
Mn1	0.0350 (2)	0.0365 (2)	0.0407 (2)	−0.0010 (2)	0.00912 (18)	0.0005 (2)
N1	0.0405 (15)	0.0371 (14)	0.0532 (17)	0.0025 (13)	0.0059 (14)	−0.0013 (12)
N2	0.0407 (17)	0.0572 (17)	0.0521 (17)	0.0059 (15)	0.0158 (14)	0.0105 (14)
N3	0.0369 (15)	0.0316 (12)	0.0466 (15)	0.0019 (12)	0.0108 (12)	0.0025 (11)
N4	0.0351 (14)	0.0355 (13)	0.0349 (13)	0.0010 (11)	0.0044 (11)	−0.0026 (10)
N5	0.0391 (15)	0.0414 (14)	0.0392 (14)	−0.0022 (13)	0.0108 (13)	−0.0024 (12)
N6	0.0385 (16)	0.0507 (15)	0.0478 (16)	−0.0003 (13)	0.0074 (14)	0.0038 (13)
C1	0.049 (2)	0.0463 (19)	0.072 (3)	0.0035 (18)	−0.002 (2)	0.0037 (17)
C2	0.051 (2)	0.0386 (19)	0.082 (3)	0.0042 (17)	0.014 (2)	0.0030 (18)
C3	0.060 (2)	0.0408 (18)	0.065 (2)	−0.0132 (18)	0.019 (2)	−0.0115 (17)
C4	0.042 (2)	0.051 (2)	0.049 (2)	−0.0118 (17)	0.0085 (16)	−0.0028 (16)
C5	0.0375 (18)	0.0406 (16)	0.0423 (18)	−0.0037 (14)	0.0153 (15)	0.0026 (14)
C6	0.0324 (18)	0.0448 (18)	0.059 (2)	−0.0030 (15)	0.0046 (16)	0.0072 (16)
C7	0.045 (2)	0.080 (3)	0.056 (2)	0.015 (2)	0.0142 (19)	0.017 (2)
C8	0.059 (3)	0.081 (3)	0.070 (3)	0.012 (2)	0.034 (2)	0.017 (2)
C9	0.046 (2)	0.067 (2)	0.077 (3)	0.015 (2)	0.026 (2)	0.015 (2)
C10	0.046 (2)	0.060 (2)	0.062 (2)	0.0140 (19)	0.0116 (19)	0.0116 (19)
C11	0.0425 (19)	0.0372 (16)	0.055 (2)	0.0008 (16)	0.0143 (17)	0.0022 (15)
C12	0.045 (2)	0.0434 (17)	0.055 (2)	0.0059 (16)	0.0163 (17)	0.0098 (16)
C13	0.0408 (18)	0.0341 (15)	0.0421 (17)	0.0045 (15)	0.0059 (15)	−0.0008 (13)
C14	0.049 (2)	0.0505 (19)	0.0447 (19)	−0.0019 (17)	0.0003 (16)	0.0024 (16)
C15	0.068 (3)	0.052 (2)	0.0386 (18)	0.008 (2)	0.0013 (17)	0.0047 (16)
C16	0.069 (3)	0.059 (2)	0.0304 (17)	0.000 (2)	0.0076 (17)	0.0110 (16)
C17	0.050 (2)	0.055 (2)	0.0418 (18)	−0.0016 (18)	0.0128 (16)	0.0034 (16)
C18	0.0410 (18)	0.0382 (16)	0.0342 (16)	0.0002 (14)	0.0088 (14)	0.0016 (13)
C19	0.048 (2)	0.057 (2)	0.0443 (19)	0.0036 (18)	0.0127 (16)	−0.0091 (16)
C20	0.069 (3)	0.063 (2)	0.046 (2)	0.006 (2)	0.007 (2)	−0.0137 (18)
C21	0.112 (4)	0.046 (2)	0.058 (2)	−0.016 (2)	0.017 (3)	−0.0156 (19)
C22	0.090 (3)	0.054 (2)	0.049 (2)	−0.023 (2)	0.018 (2)	−0.0054 (18)
C23	0.0395 (18)	0.0443 (17)	0.0334 (16)	−0.0040 (15)	0.0042 (14)	−0.0013 (13)
C24	0.0388 (19)	0.0471 (19)	0.0436 (18)	−0.0075 (16)	0.0111 (15)	−0.0021 (14)
C25	0.048 (2)	0.059 (2)	0.049 (2)	−0.0088 (19)	0.0076 (18)	0.0085 (17)
C26	0.050 (2)	0.052 (2)	0.064 (2)	0.0024 (19)	−0.005 (2)	0.0071 (18)
C27	0.049 (2)	0.053 (2)	0.077 (3)	0.0140 (19)	0.007 (2)	−0.005 (2)
C28	0.043 (2)	0.062 (2)	0.061 (2)	0.0078 (19)	0.0129 (18)	−0.0063 (19)
C29	0.0392 (19)	0.0437 (18)	0.0454 (18)	0.0006 (16)	0.0096 (15)	−0.0058 (15)
C30	0.042 (2)	0.0504 (18)	0.0384 (17)	0.0046 (16)	0.0093 (15)	−0.0037 (14)
O1	0.0731 (19)	0.0666 (17)	0.0601 (15)	0.0186 (15)	0.0301 (15)	0.0251 (13)
O2	0.150 (4)	0.070 (2)	0.134 (3)	0.035 (2)	0.079 (3)	0.039 (2)
Cl1A	0.074 (3)	0.0625 (15)	0.085 (3)	0.0218 (18)	0.045 (2)	0.0324 (15)
O3A	0.191 (15)	0.126 (9)	0.071 (5)	0.033 (9)	0.074 (8)	0.024 (5)
O4A	0.075 (5)	0.094 (5)	0.233 (13)	−0.011 (4)	0.045 (7)	0.043 (7)
Cl1B	0.088 (5)	0.120 (5)	0.082 (4)	0.040 (4)	0.050 (4)	0.058 (4)
O3B	0.21 (2)	0.29 (2)	0.089 (10)	−0.086 (19)	−0.047 (13)	0.089 (12)
O4B	0.16 (2)	0.140 (16)	0.21 (3)	0.044 (14)	0.16 (2)	0.090 (17)
Cl2	0.0750 (8)	0.0931 (8)	0.0693 (7)	0.0072 (7)	0.0246 (6)	0.0060 (6)
O5	0.187 (5)	0.102 (3)	0.096 (3)	0.002 (3)	0.022 (3)	0.020 (2)
O6	0.103 (3)	0.179 (4)	0.121 (3)	−0.022 (3)	0.057 (3)	−0.033 (3)
O7	0.325 (12)	0.194 (6)	0.124 (4)	−0.119 (7)	0.028 (5)	−0.049 (4)
O8	0.176 (6)	0.116 (4)	0.366 (10)	0.064 (4)	0.184 (7)	0.098 (5)

### Geometric parameters (Å, °)

**Table:**

Mn1—N5	2.273 (3)	C14—H14A	0.9700
Mn1—N1	2.294 (3)	C14—H14B	0.9700
Mn1—O1	2.331 (3)	C15—C16	1.487 (6)
Mn1—N4	2.388 (3)	C15—H15A	0.9700
Mn1—N2	2.390 (3)	C15—H15B	0.9700
Mn1—N6	2.396 (3)	C16—C17	1.527 (5)
Mn1—N3	2.422 (3)	C16—H16A	0.9700
N1—C5	1.338 (4)	C16—H16B	0.9700
N1—C1	1.356 (4)	C17—C18	1.548 (5)
N2—C11	1.334 (4)	C17—H17A	0.9700
N2—C7	1.334 (5)	C17—H17B	0.9700
N3—C12	1.478 (4)	C18—H18	0.9800
N3—C6	1.483 (4)	C19—C20	1.355 (5)
N3—C13	1.495 (4)	C19—H19	0.9300
N4—C24	1.470 (4)	C20—C21	1.364 (6)
N4—C30	1.475 (4)	C20—H20	0.9300
N4—C18	1.505 (4)	C21—C22	1.377 (6)
N5—C23	1.329 (4)	C21—H21	0.9300
N5—C19	1.353 (4)	C22—C23	1.381 (5)
N6—C25	1.335 (4)	C22—H22	0.9300
N6—C29	1.342 (4)	C23—C24	1.505 (5)
C1—C2	1.380 (5)	C24—H24A	0.9700
C1—H1	0.9300	C24—H24B	0.9700
C2—C3	1.365 (6)	C25—C26	1.378 (6)
C2—H2	0.9300	C25—H25	0.9300
C3—C4	1.378 (5)	C26—C27	1.361 (6)
C3—H3	0.9300	C26—H26	0.9300
C4—C5	1.378 (5)	C27—C28	1.369 (5)
C4—H4	0.9300	C27—H27	0.9300
C5—C6	1.497 (5)	C28—C29	1.377 (5)
C6—H6A	0.9700	C28—H28	0.9300
C6—H6B	0.9700	C29—C30	1.491 (5)
C7—C8	1.371 (6)	C30—H30A	0.9700
C7—H7	0.9300	C30—H30B	0.9700
C8—C9	1.374 (6)	O1—Cl1B	1.437 (7)
C8—H8	0.9300	O1—Cl1A	1.457 (5)
C9—C10	1.367 (5)	O2—Cl1A	1.454 (7)
C9—H9	0.9300	O2—Cl1B	1.456 (11)
C10—C11	1.378 (5)	Cl1A—O4A	1.415 (11)
C10—H10	0.9300	Cl1A—O3A	1.437 (12)
C11—C12	1.517 (5)	Cl1B—O3B	1.30 (2)
C12—H12A	0.9700	Cl1B—O4B	1.369 (16)
C12—H12B	0.9700	Cl2—O7	1.327 (5)
C13—C18	1.520 (5)	Cl2—O5	1.395 (4)
C13—C14	1.536 (5)	Cl2—O8	1.419 (5)
C13—H13	0.9800	Cl2—O6	1.439 (5)
C14—C15	1.512 (5)		
			
N5—Mn1—N1	176.02 (11)	C15—C14—C13	111.2 (3)
N5—Mn1—O1	89.66 (10)	C15—C14—H14A	109.4
N1—Mn1—O1	90.69 (11)	C13—C14—H14A	109.4
N5—Mn1—N4	73.98 (9)	C15—C14—H14B	109.4
N1—Mn1—N4	103.60 (10)	C13—C14—H14B	109.4
O1—Mn1—N4	144.44 (10)	H14A—C14—H14B	108.0
N5—Mn1—N2	84.18 (10)	C16—C15—C14	109.6 (3)
N1—Mn1—N2	99.77 (11)	C16—C15—H15A	109.8
O1—Mn1—N2	78.89 (10)	C14—C15—H15A	109.8
N4—Mn1—N2	128.73 (9)	C16—C15—H15B	109.8
N5—Mn1—N6	90.22 (10)	C14—C15—H15B	109.8
N1—Mn1—N6	85.98 (10)	H15A—C15—H15B	108.2
O1—Mn1—N6	78.13 (10)	C15—C16—C17	110.2 (3)
N4—Mn1—N6	70.76 (9)	C15—C16—H16A	109.6
N2—Mn1—N6	156.36 (10)	C17—C16—H16A	109.6
N5—Mn1—N3	109.95 (9)	C15—C16—H16B	109.6
N1—Mn1—N3	72.01 (9)	C17—C16—H16B	109.6
O1—Mn1—N3	141.38 (10)	H16A—C16—H16B	108.1
N4—Mn1—N3	74.13 (9)	C16—C17—C18	111.0 (3)
N2—Mn1—N3	70.80 (10)	C16—C17—H17A	109.4
N6—Mn1—N3	132.33 (10)	C18—C17—H17A	109.4
C5—N1—C1	117.7 (3)	C16—C17—H17B	109.4
C5—N1—Mn1	118.9 (2)	C18—C17—H17B	109.4
C1—N1—Mn1	123.3 (2)	H17A—C17—H17B	108.0
C11—N2—C7	117.3 (3)	N4—C18—C13	111.6 (2)
C11—N2—Mn1	115.4 (2)	N4—C18—C17	112.6 (3)
C7—N2—Mn1	127.0 (2)	C13—C18—C17	110.8 (3)
C12—N3—C6	108.9 (3)	N4—C18—H18	107.2
C12—N3—C13	113.0 (2)	C13—C18—H18	107.2
C6—N3—C13	110.2 (2)	C17—C18—H18	107.2
C12—N3—Mn1	106.0 (2)	N5—C19—C20	122.3 (4)
C6—N3—Mn1	107.98 (18)	N5—C19—H19	118.8
C13—N3—Mn1	110.59 (19)	C20—C19—H19	118.8
C24—N4—C30	110.1 (3)	C19—C20—C21	119.8 (4)
C24—N4—C18	110.1 (2)	C19—C20—H20	120.1
C30—N4—C18	112.9 (2)	C21—C20—H20	120.1
C24—N4—Mn1	108.93 (17)	C20—C21—C22	118.6 (4)
C30—N4—Mn1	103.21 (19)	C20—C21—H21	120.7
C18—N4—Mn1	111.38 (19)	C22—C21—H21	120.7
C23—N5—C19	118.0 (3)	C21—C22—C23	119.2 (4)
C23—N5—Mn1	117.7 (2)	C21—C22—H22	120.4
C19—N5—Mn1	124.2 (2)	C23—C22—H22	120.4
C25—N6—C29	117.7 (3)	N5—C23—C22	122.0 (3)
C25—N6—Mn1	129.5 (2)	N5—C23—C24	118.3 (3)
C29—N6—Mn1	112.7 (2)	C22—C23—C24	119.7 (3)
N1—C1—C2	122.9 (4)	N4—C24—C23	114.5 (3)
N1—C1—H1	118.5	N4—C24—H24A	108.6
C2—C1—H1	118.5	C23—C24—H24A	108.6
C3—C2—C1	118.9 (3)	N4—C24—H24B	108.6
C3—C2—H2	120.6	C23—C24—H24B	108.6
C1—C2—H2	120.6	H24A—C24—H24B	107.6
C2—C3—C4	118.6 (3)	N6—C25—C26	123.1 (4)
C2—C3—H3	120.7	N6—C25—H25	118.4
C4—C3—H3	120.7	C26—C25—H25	118.4
C5—C4—C3	120.4 (3)	C27—C26—C25	118.8 (4)
C5—C4—H4	119.8	C27—C26—H26	120.6
C3—C4—H4	119.8	C25—C26—H26	120.6
N1—C5—C4	121.5 (3)	C26—C27—C28	118.7 (4)
N1—C5—C6	116.9 (3)	C26—C27—H27	120.7
C4—C5—C6	121.4 (3)	C28—C27—H27	120.7
N3—C6—C5	112.7 (3)	C27—C28—C29	120.1 (4)
N3—C6—H6A	109.0	C27—C28—H28	119.9
C5—C6—H6A	109.0	C29—C28—H28	119.9
N3—C6—H6B	109.0	N6—C29—C28	121.5 (3)
C5—C6—H6B	109.0	N6—C29—C30	117.3 (3)
H6A—C6—H6B	107.8	C28—C29—C30	121.2 (3)
N2—C7—C8	123.6 (4)	N4—C30—C29	111.5 (3)
N2—C7—H7	118.2	N4—C30—H30A	109.3
C8—C7—H7	118.2	C29—C30—H30A	109.3
C7—C8—C9	118.4 (4)	N4—C30—H30B	109.3
C7—C8—H8	120.8	C29—C30—H30B	109.3
C9—C8—H8	120.8	H30A—C30—H30B	108.0
C10—C9—C8	118.7 (4)	Cl1B—O1—Mn1	162.9 (5)
C10—C9—H9	120.7	Cl1A—O1—Mn1	143.4 (4)
C8—C9—H9	120.7	O4A—Cl1A—O3A	114.4 (9)
C9—C10—C11	119.5 (4)	O4A—Cl1A—O2	111.0 (6)
C9—C10—H10	120.2	O3A—Cl1A—O2	109.7 (7)
C11—C10—H10	120.2	O4A—Cl1A—O1	110.2 (5)
N2—C11—C10	122.4 (3)	O3A—Cl1A—O1	104.7 (7)
N2—C11—C12	117.0 (3)	O2—Cl1A—O1	106.4 (4)
C10—C11—C12	120.6 (3)	O3B—Cl1B—O4B	115.7 (16)
N3—C12—C11	109.8 (3)	O3B—Cl1B—O1	112.6 (11)
N3—C12—H12A	109.7	O4B—Cl1B—O1	111.1 (11)
C11—C12—H12A	109.7	O3B—Cl1B—O2	107.4 (13)
N3—C12—H12B	109.7	O4B—Cl1B—O2	101.7 (13)
C11—C12—H12B	109.7	O1—Cl1B—O2	107.4 (6)
H12A—C12—H12B	108.2	O7—Cl2—O5	110.0 (3)
N3—C13—C18	111.3 (2)	O7—Cl2—O8	109.4 (6)
N3—C13—C14	114.1 (3)	O5—Cl2—O8	103.3 (4)
C18—C13—C14	110.6 (3)	O7—Cl2—O6	117.0 (4)
N3—C13—H13	106.8	O5—Cl2—O6	116.2 (3)
C18—C13—H13	106.8	O8—Cl2—O6	99.2 (3)
C14—C13—H13	106.8		
			
O1—Mn1—N1—C5	−135.9 (3)	C7—C8—C9—C10	1.5 (7)
N4—Mn1—N1—C5	77.0 (3)	C8—C9—C10—C11	−0.9 (6)
N2—Mn1—N1—C5	−57.0 (3)	C7—N2—C11—C10	3.0 (5)
N6—Mn1—N1—C5	146.1 (3)	Mn1—N2—C11—C10	−171.1 (3)
N3—Mn1—N1—C5	9.0 (2)	C7—N2—C11—C12	−175.9 (3)
O1—Mn1—N1—C1	47.9 (3)	Mn1—N2—C11—C12	9.9 (4)
N4—Mn1—N1—C1	−99.2 (3)	C9—C10—C11—N2	−1.4 (6)
N2—Mn1—N1—C1	126.7 (3)	C9—C10—C11—C12	177.5 (4)
N6—Mn1—N1—C1	−30.2 (3)	C6—N3—C12—C11	−66.4 (3)
N3—Mn1—N1—C1	−167.3 (3)	C13—N3—C12—C11	170.8 (3)
N5—Mn1—N2—C11	−100.2 (2)	Mn1—N3—C12—C11	49.6 (3)
N1—Mn1—N2—C11	80.2 (2)	N2—C11—C12—N3	−41.9 (4)
O1—Mn1—N2—C11	169.0 (3)	C10—C11—C12—N3	139.1 (3)
N4—Mn1—N2—C11	−36.2 (3)	C12—N3—C13—C18	−79.4 (3)
N6—Mn1—N2—C11	−177.3 (2)	C6—N3—C13—C18	158.6 (3)
N3—Mn1—N2—C11	13.3 (2)	Mn1—N3—C13—C18	39.3 (3)
N5—Mn1—N2—C7	86.3 (3)	C12—N3—C13—C14	46.8 (4)
N1—Mn1—N2—C7	−93.3 (3)	C6—N3—C13—C14	−75.3 (3)
O1—Mn1—N2—C7	−4.5 (3)	Mn1—N3—C13—C14	165.4 (2)
N4—Mn1—N2—C7	150.3 (3)	N3—C13—C14—C15	177.1 (3)
N6—Mn1—N2—C7	9.2 (5)	C18—C13—C14—C15	−56.4 (4)
N3—Mn1—N2—C7	−160.2 (3)	C13—C14—C15—C16	60.4 (4)
N5—Mn1—N3—C12	43.0 (2)	C14—C15—C16—C17	−61.0 (4)
N1—Mn1—N3—C12	−140.7 (2)	C15—C16—C17—C18	58.3 (4)
O1—Mn1—N3—C12	−73.4 (2)	C24—N4—C18—C13	159.6 (2)
N4—Mn1—N3—C12	108.9 (2)	C30—N4—C18—C13	−76.9 (3)
N2—Mn1—N3—C12	−33.11 (19)	Mn1—N4—C18—C13	38.6 (3)
N6—Mn1—N3—C12	152.61 (19)	C24—N4—C18—C17	−75.1 (3)
N5—Mn1—N3—C6	159.5 (2)	C30—N4—C18—C17	48.3 (3)
N1—Mn1—N3—C6	−24.1 (2)	Mn1—N4—C18—C17	163.9 (2)
O1—Mn1—N3—C6	43.2 (3)	N3—C13—C18—N4	−52.9 (3)
N4—Mn1—N3—C6	−134.6 (2)	C14—C13—C18—N4	179.0 (3)
N2—Mn1—N3—C6	83.5 (2)	N3—C13—C18—C17	−179.2 (3)
N6—Mn1—N3—C6	−90.8 (2)	C14—C13—C18—C17	52.7 (4)
N5—Mn1—N3—C13	−79.9 (2)	C16—C17—C18—N4	−179.8 (3)
N1—Mn1—N3—C13	96.5 (2)	C16—C17—C18—C13	−54.1 (4)
O1—Mn1—N3—C13	163.77 (18)	C23—N5—C19—C20	−2.1 (5)
N4—Mn1—N3—C13	−13.95 (18)	Mn1—N5—C19—C20	174.6 (3)
N2—Mn1—N3—C13	−155.9 (2)	N5—C19—C20—C21	0.2 (6)
N6—Mn1—N3—C13	29.8 (2)	C19—C20—C21—C22	1.4 (7)
N5—Mn1—N4—C24	−17.8 (2)	C20—C21—C22—C23	−1.1 (7)
N1—Mn1—N4—C24	158.9 (2)	C19—N5—C23—C22	2.5 (5)
O1—Mn1—N4—C24	47.9 (3)	Mn1—N5—C23—C22	−174.5 (3)
N2—Mn1—N4—C24	−86.4 (2)	C19—N5—C23—C24	−174.9 (3)
N6—Mn1—N4—C24	78.2 (2)	Mn1—N5—C23—C24	8.1 (4)
N3—Mn1—N4—C24	−134.6 (2)	C21—C22—C23—N5	−0.9 (6)
N5—Mn1—N4—C30	−134.8 (2)	C21—C22—C23—C24	176.4 (4)
N1—Mn1—N4—C30	41.9 (2)	C30—N4—C24—C23	140.4 (3)
O1—Mn1—N4—C30	−69.1 (2)	C18—N4—C24—C23	−94.5 (3)
N2—Mn1—N4—C30	156.64 (18)	Mn1—N4—C24—C23	27.9 (3)
N6—Mn1—N4—C30	−38.81 (18)	N5—C23—C24—N4	−25.6 (4)
N3—Mn1—N4—C30	108.42 (19)	C22—C23—C24—N4	157.0 (3)
N5—Mn1—N4—C18	103.79 (19)	C29—N6—C25—C26	0.2 (5)
N1—Mn1—N4—C18	−79.47 (19)	Mn1—N6—C25—C26	−177.5 (3)
O1—Mn1—N4—C18	169.45 (19)	N6—C25—C26—C27	−0.7 (6)
N2—Mn1—N4—C18	35.2 (2)	C25—C26—C27—C28	0.2 (6)
N6—Mn1—N4—C18	−160.2 (2)	C26—C27—C28—C29	0.7 (6)
N3—Mn1—N4—C18	−12.99 (17)	C25—N6—C29—C28	0.8 (5)
O1—Mn1—N5—C23	−142.3 (2)	Mn1—N6—C29—C28	178.9 (3)
N4—Mn1—N5—C23	5.7 (2)	C25—N6—C29—C30	179.8 (3)
N2—Mn1—N5—C23	138.8 (2)	Mn1—N6—C29—C30	−2.2 (4)
N6—Mn1—N5—C23	−64.2 (2)	C27—C28—C29—N6	−1.3 (5)
N3—Mn1—N5—C23	71.7 (3)	C27—C28—C29—C30	179.8 (3)
O1—Mn1—N5—C19	40.9 (3)	C24—N4—C30—C29	−63.3 (3)
N4—Mn1—N5—C19	−171.1 (3)	C18—N4—C30—C29	173.2 (3)
N2—Mn1—N5—C19	−38.0 (3)	Mn1—N4—C30—C29	52.8 (3)
N6—Mn1—N5—C19	119.0 (3)	N6—C29—C30—N4	−36.1 (4)
N3—Mn1—N5—C19	−105.1 (3)	C28—C29—C30—N4	142.8 (3)
N5—Mn1—N6—C25	−85.9 (3)	N5—Mn1—O1—Cl1B	153 (2)
N1—Mn1—N6—C25	95.3 (3)	N1—Mn1—O1—Cl1B	−23 (2)
O1—Mn1—N6—C25	3.7 (3)	N4—Mn1—O1—Cl1B	91 (2)
N4—Mn1—N6—C25	−158.8 (3)	N2—Mn1—O1—Cl1B	−123 (2)
N2—Mn1—N6—C25	−10.0 (5)	N6—Mn1—O1—Cl1B	62 (2)
N3—Mn1—N6—C25	156.4 (3)	N3—Mn1—O1—Cl1B	−85 (2)
N5—Mn1—N6—C29	96.3 (2)	N5—Mn1—O1—Cl1A	−167.8 (5)
N1—Mn1—N6—C29	−82.5 (2)	N1—Mn1—O1—Cl1A	16.2 (5)
O1—Mn1—N6—C29	−174.0 (2)	N4—Mn1—O1—Cl1A	131.1 (5)
N4—Mn1—N6—C29	23.4 (2)	N2—Mn1—O1—Cl1A	−83.6 (5)
N2—Mn1—N6—C29	172.2 (2)	N6—Mn1—O1—Cl1A	101.9 (5)
N3—Mn1—N6—C29	−21.4 (3)	N3—Mn1—O1—Cl1A	−45.1 (5)
C5—N1—C1—C2	−1.0 (6)	Cl1B—O2—Cl1A—O4A	169.6 (13)
Mn1—N1—C1—C2	175.3 (3)	Cl1B—O2—Cl1A—O3A	42.3 (12)
N1—C1—C2—C3	−0.3 (7)	Cl1B—O2—Cl1A—O1	−70.5 (9)
C1—C2—C3—C4	1.0 (6)	Cl1B—O1—Cl1A—O4A	−167.6 (15)
C2—C3—C4—C5	−0.4 (6)	Mn1—O1—Cl1A—O4A	38.2 (9)
C1—N1—C5—C4	1.6 (5)	Cl1B—O1—Cl1A—O3A	−44.2 (12)
Mn1—N1—C5—C4	−174.9 (2)	Mn1—O1—Cl1A—O3A	161.6 (7)
C1—N1—C5—C6	−174.8 (3)	Cl1B—O1—Cl1A—O2	72.0 (12)
Mn1—N1—C5—C6	8.8 (4)	Mn1—O1—Cl1A—O2	−82.2 (8)
C3—C4—C5—N1	−0.9 (5)	Cl1A—O1—Cl1B—O3B	169 (2)
C3—C4—C5—C6	175.3 (3)	Mn1—O1—Cl1B—O3B	−129 (3)
C12—N3—C6—C5	151.6 (3)	Cl1A—O1—Cl1B—O4B	37.8 (14)
C13—N3—C6—C5	−83.9 (3)	Mn1—O1—Cl1B—O4B	100 (2)
Mn1—N3—C6—C5	37.0 (3)	Cl1A—O1—Cl1B—O2	−72.6 (15)
N1—C5—C6—N3	−32.3 (4)	Mn1—O1—Cl1B—O2	−11 (3)
C4—C5—C6—N3	151.4 (3)	Cl1A—O2—Cl1B—O3B	−164.7 (16)
C11—N2—C7—C8	−2.4 (6)	Cl1A—O2—Cl1B—O4B	−42.8 (14)
Mn1—N2—C7—C8	171.0 (3)	Cl1A—O2—Cl1B—O1	74.0 (11)
N2—C7—C8—C9	0.2 (7)		

### Hydrogen-bond geometry (Å, °)

**Table:**

*D*—H···*A*	*D*—H	H···*A*	*D*···*A*	*D*—H···*A*
C7—H7···O1	0.93	2.41	3.006 (5)	122
C17—H17A···O2^i^	0.97	2.48	3.413 (5)	161
C18—H18···O8^ii^	0.98	2.47	3.436 (6)	170
C20—H20···O6	0.93	2.46	3.347 (6)	159
C25—H25···O1	0.93	2.46	3.038 (5)	121
C25—H25···O2	0.93	2.51	3.199 (5)	131
C28—H28···O8^iii^	0.93	2.48	3.243 (6)	139
C30—H30A···O3A^i^	0.97	2.55	3.434 (11)	152

Symmetry codes: (i) *x*, −*y*+1, *z*+1/2; (ii) *x*, −*y*, *z*+1/2; (iii) *x*−1/2, −*y*+1/2, *z*+1/2.
